# Angiomatoid fibrous histiocytoma: a series of seven cases including genetically confirmed aggressive cases and a literature review

**DOI:** 10.1186/s12891-017-1390-y

**Published:** 2017-01-23

**Authors:** Kenichi Saito, Eisuke Kobayashi, Akihiko Yoshida, Yoshihiro Araki, Daisuke Kubota, Yoshikazu Tanzawa, Akira Kawai, Takashi Yanagawa, Kenji Takagishi, Hirokazu Chuman

**Affiliations:** 1 Division of Muscloskeletal Oncology, National Cancer Center Hospital, 5-5-1 Tsukiji, Chuo-ku, Tokyo, 104-0045 Japan; 2 Division of Pathology and Clinical Laboratories, National Cancer Center Hospital, 5-5-1 Tsukiji, Chuo-ku, Tokyo, 104-0045 Japan; 3 Department of Orthopaedic Surgery, Gunma University Graduate School of Medicine, 3-39-22 Showa-machi, Maebashi, Gunma 371-8511 Japan

**Keywords:** Angiomatoid fibrous histiocytoma, Diagnosis, Prognosis

## Abstract

**Background:**

Angiomatoid fibrous histiocytoma (AFH) is a rare soft tissue tumor of intermediate biologic potential. Because of its rarity and nonspecific radiological and diverse pathological findings, AFH is often clinically misdiagnosed. However, few clinical reports have described this tumor. As reported herein, we analyzed the clinical and radiological features and clinical outcomes of AFH.

**Methods:**

We retrospectively reviewed the medical records of seven cases histopathologically diagnosed as AFH. We examined clinical features, MRI findings, histopathological diagnoses, treatments, and outcomes.

**Results:**

These seven cases comprised five male and two female patients with ages ranging from 8 to 50 years old. The primary locations included upper extremities in 2, lower extremities in 4, and the inguinal region in one patient. Of the tumors, 4 occurred in subcutaneous tissues and 3 occurred in deep tissues. No cases were diagnosed as AFH from MRI and needle biopsy results. All cases were diagnosed histopathologically after excision. After treatment, 2 patients (29%) had tumor recurrence and metastasis, one of whom died from disease progression. These 2 aggressive cases involved both EWSR1 and CREB1 gene rearrangements as determined by FISH. The other patients were alive and well without recurrence or metastasis.

**Conclusion:**

AFH is a rare tumor that is difficult to diagnose. Therefore, it tends to be misdiagnosed and to be treated inadequately by referring physicians. Surgeons must therefore be mindful of the presence of AFH, learn about appropriate treatment necessary for this tumor, and conduct careful follow-up because AFH can engender poor outcomes.

## Background

Angiomatoid fibrous histiocytoma (AFH), a rare soft tissue neoplasm, was described initially as “angiomatoid malignant fibrous histiocytoma” by Enzinger in 1979 [1]. Today, the precise line of differentiation remains unknown, but this entity is no longer regarded as “malignant” because of its benign microscopic appearance and favorable prognosis. In the 2013 World Health Organization (WHO) classification, this tumor was placed under the category of “intermediate tumors of uncertain differentiation” as AFH [2]. AFH often presents as a soft tissue mass in the subcutis or deep dermis in the extremities of children and young adults, with a median age of 13 years [1, 3, 4]. Clinically and radiologically, it is often difficult to differentiate this tumor from vascular tumors, such as hemangioendothelioma and angiosarcoma, or simply organized hematoma. Although the prognosis of patients with AFH is not poor, it recurs in up to 15% of cases and metastasizes in fewer than 1% of cases [5, 6]. Because of its rarity, few reports have described its related clinical and radiological findings or treatment outcomes. This report presents the (i) clinical features, (ii) magnetic resonance imaging (MRI) findings, (iii) histopathological diagnoses, and (iv) treatments and outcomes of seven cases of AFH.

## Methods

**Fig. 1:**
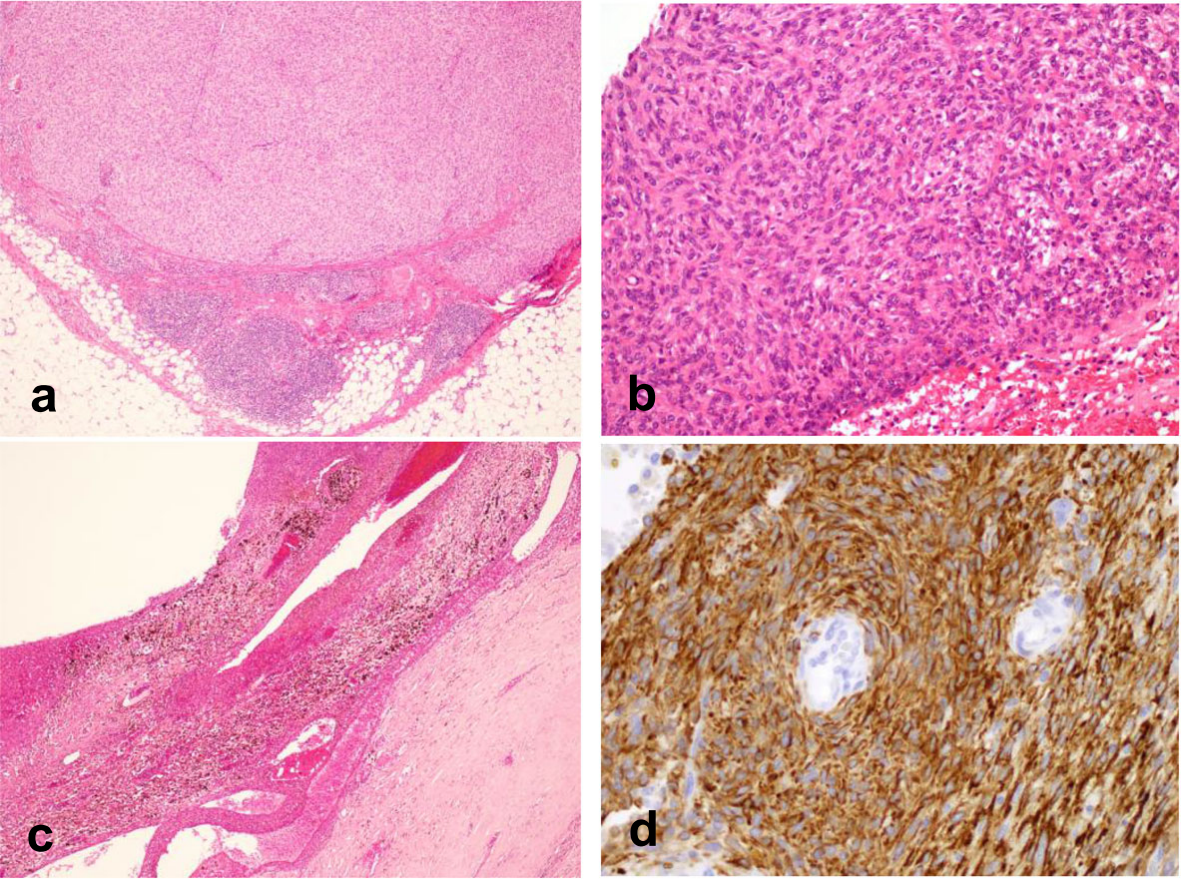
Histopathological studies of AFH. The photomicrographic images show: (**a**) peritumoral lymphoplasmacytic cuffing mimicking a lymph node; (**b**) the growth of spindle cells with storiform distributions; (**c**) pseudoangiomatous spaces filled with blood, fibrous pseudocapsules, and hemosiderin deposition; and (**d**) desmin immunoreactivity. (**a**) Case 3 and (**b**)-(**d**) case 6

**Fig. 2:**
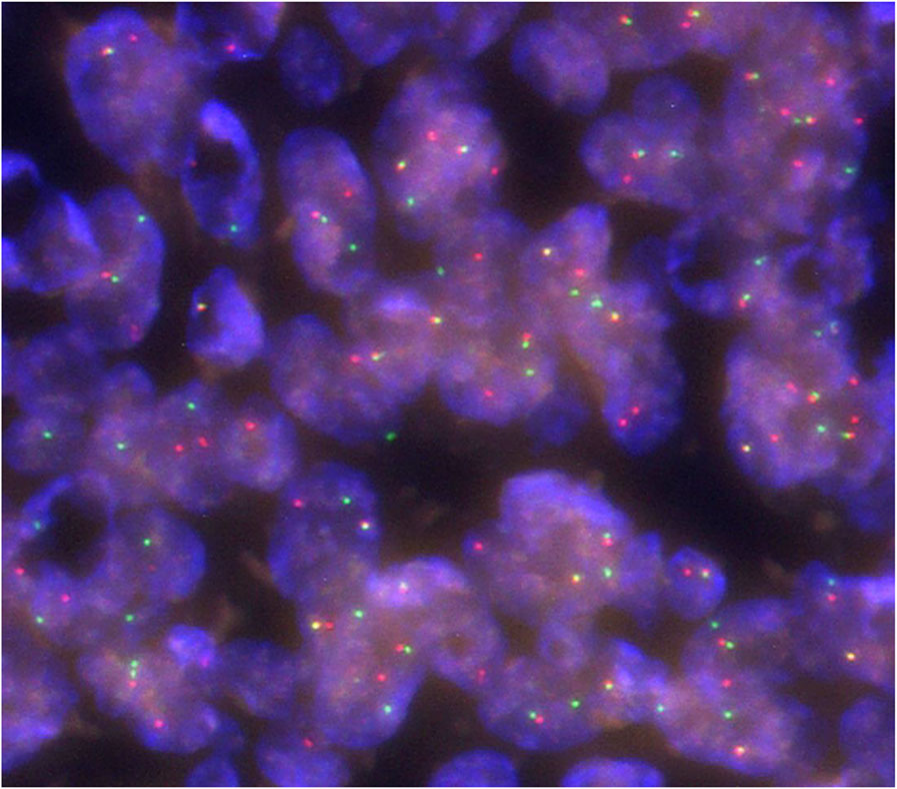
A FISH analysis of AFH. The presence of split red and green signals indicates the presence of *EWSR1* rearrangement (case 6)

We retrospectively reviewed the medical records of seven cases histopathologically diagnosed as AFH at two institutions. All histopathological specimens were examined by a soft tissue pathologist to confirm the diagnosis (Figs. 1 and 2). All diagnoses were classified according to current WHO criteria [2]. All of the patients provided informed consent to the publication of case information. In the case of children, who could not give consent, their parents provided consent on their behalf.

Clinical features related to gender, age, tumor size and site, and symptoms were obtained (Table 1). Furthermore, we examined the MRI findings (Table 2), histopathological diagnoses, treatments, and outcomes (Table 3).

**Table 1:** Clinical features of patients with AFH

Case No.	Sex	Age (years)	Size (mm)	Site	Symptom
1	F	28	23	thigh, subcutaneous	mass
2	M	50	45	finger, subcutaneous	mass
3	F	8	10	crus, subcutaneous	mass, pain
4	M	21	30	upper arm, subcutaneous	mass
5	M	36	45	popliteus, intermuscular	no
6	M	34	303	thigh, intramuscular	mass
7	M	8	62	groin, intramuscular	mass

**Table 2:** Magnetic resonance imaging findings

Case No.	T1	T2	Cystic area	Multilocular area	Pseudocapsule	Fluid–fluid level	Peritumoral edema	Enhancement	Initial diagnosis
1	homo, iso to muscle	hetero	−	−	+	+	−	+	hemangioma, AVM
2	hetero, iso/hyper to muscle	hetero	−	+	−	−	+	+	NA
3	homo, iso to muscle	hetero	−	−	+	−	+	+	NA
4	homo, iso to muscle	hetero	−	+	+	−	+	+	synovial sarcoma
5	homo, iso to muscle	hetero	−	+	+	−	+	+	myxofibrosarcoma
6	hetero, iso/hyper to muscle	hetero	+	+	+	+	+	+	hemangioma, AVM
7	hetero, iso/hyper to muscle	hetero	+	+	−	−	+	NA	hematoma

*NA* not applicable, *AVM* arteriovenous malformation

**Table 3:** Histopathological diagnosis, treatments, and outcomes

Case No	Diagnosis of NB	Diagnosis of EB	Additional treatment	EWSR1 rearrangement	Recurrence / Metastasis	Outcome	Follow-up (mo.)
1	NA	AFH	WE	NA	−/−	CDF	10
2	NA	myxoid liposarcoma	ray amputation	+	−/−	CDF	5
3	Ewing sarcoma	NA	Chemo + WE	+#	+ / +	NED	18
4	spindle cell tumor	NA	WE	+	−/−	CDF	57
5	myxofibrosarcoma	NA	WE	+	−/−	CDF	61
6	NA	synovial sarcoma	Chemo + WE	+#	+ / +	DOD	55
7	NA	AFH	observation	NA	−/−	CDF	40

*NB* needle biopsy, *EB* excisional biopsy, *ME* marginal excision, *WE* wide excision, *CDF* continuous disease free, *NED* no evidence of disease, *DOD* died of disease, *NA* not applicable. #. CREB1 rearrangement was also demonstrated by FISH, using custom CREB1 break-apart probe set (GSP laboratory, Kobe, Japan)

## Results

### Clinical features

These seven cases were composed of five male and two female patients with an age range of 8 to 50 years (mean, 26.4 years). The primary locations were upper extremity in two patients, lower extremity in four patients, and the inguinal region in one patient. Regarding the tumor sites, four patients had subcutaneous lesions and three had deep lesions. Five patients presented with a painless mass, whereas one presented with a painful mass. Only one patient (case 5) had no symptoms. However, the mass had been occasionally noted as an abnormal finding after medical examinations. The average and median sizes of the tumors were 69 mm and 45 mm (range, 10–303 mm), respectively (Table 1).

### Magnetic resonance imaging

**Fig. 3:**
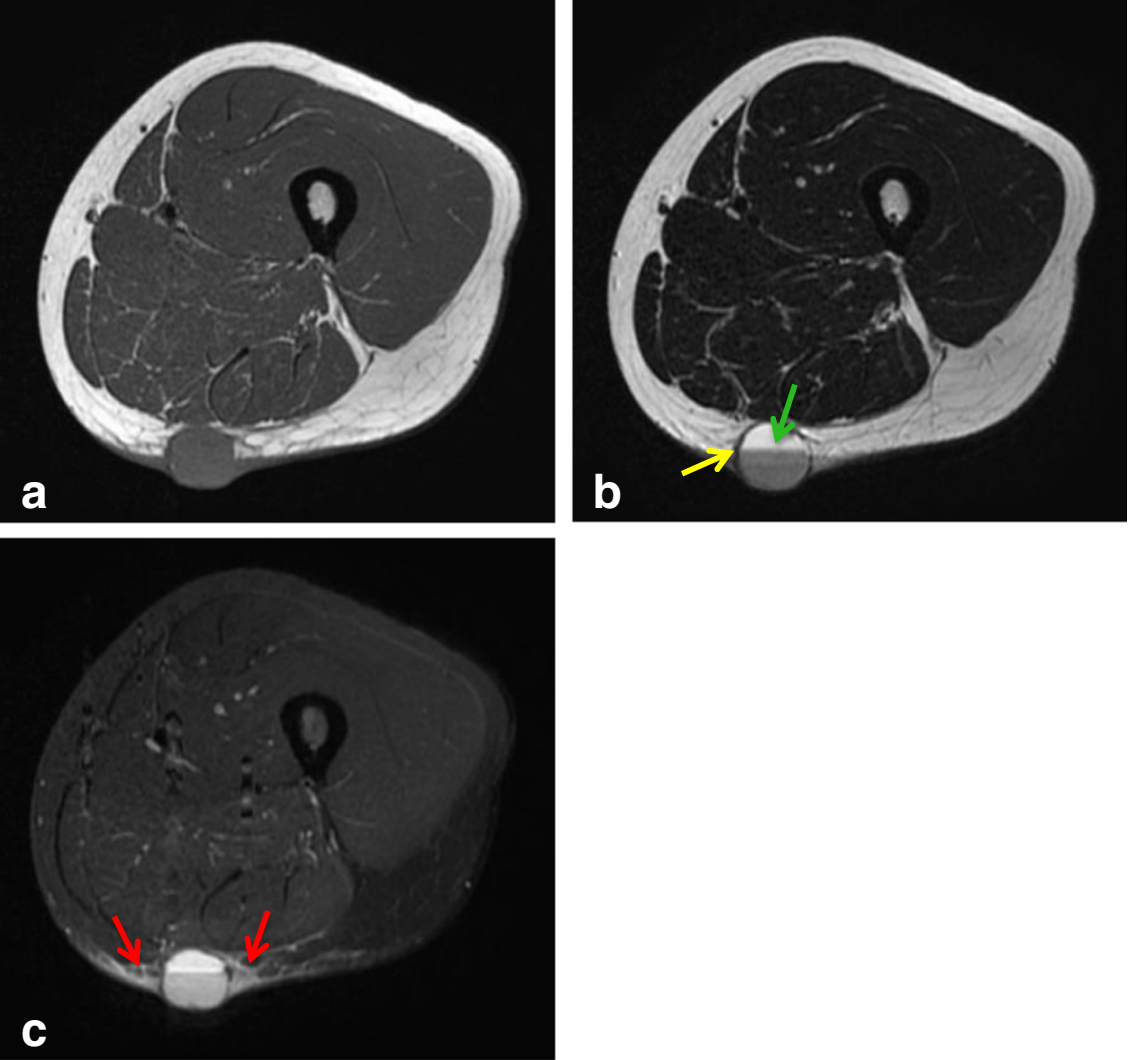
A 28-year-old woman diagnosed with angiomatoid fibrous histiocytoma (case 1): (**a**) T1-weighted spin echo, (**b**) T2-weighted spin echo, and (**c**) STIR images. A 21 × 23 × 22-mm well-circumscribed, round mass is present in the subcutaneous fat of the posterior right thigh. The lesion is homogeneously hypointense on T1 WI and presents fluid–fluid level (*green arrow*) and pseudocapsule (*yellow arrow*) on T2 WI. STIR shows peritumoral edema (*red arrows*)

**Fig. 4:**
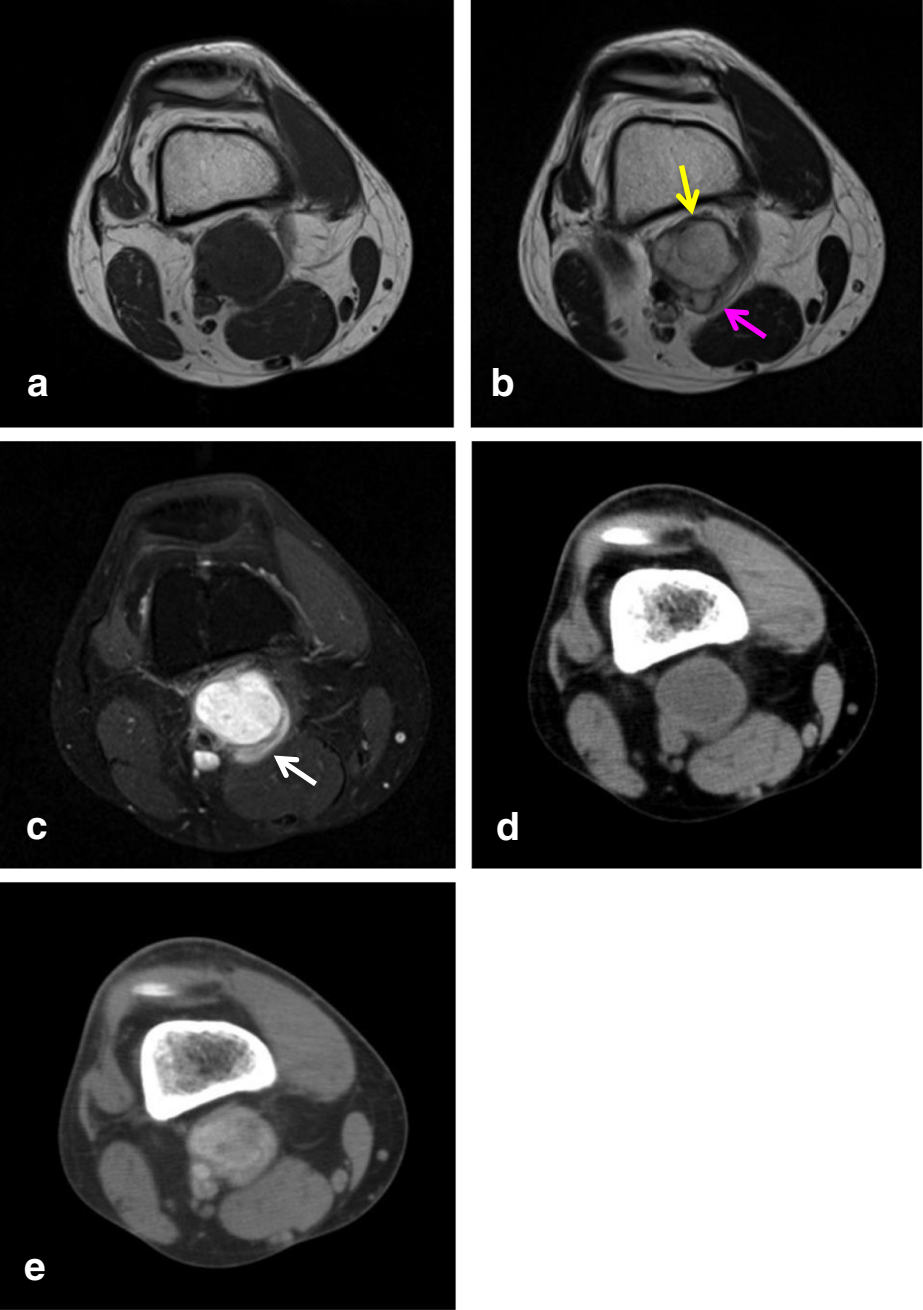
A 36-year-old man diagnosed with angiomatoid fibrous histiocytoma (case 5): (**a**) T1-weighted spin echo, (**b**) T2-weighted spin echo, and (**c**) contrast-enhanced MR images. (**d**) Non-enhanced and (**e**) enhanced CT images. A 32 × 36 × 45-mm asymptomatic mass is present in the popliteal lesion of right knee. The lesion is homogeneously isointense on T1 WI and presents with a multilocular area (pink arrow) and pseudocapsule (yellow arrow) on T2 WI. A contrast-enhanced MR image shows intratumoral and peritumoral (white arrow) enhancement. In addition, an enhanced CT image shows variegated enhancement

**Fig. 5:**
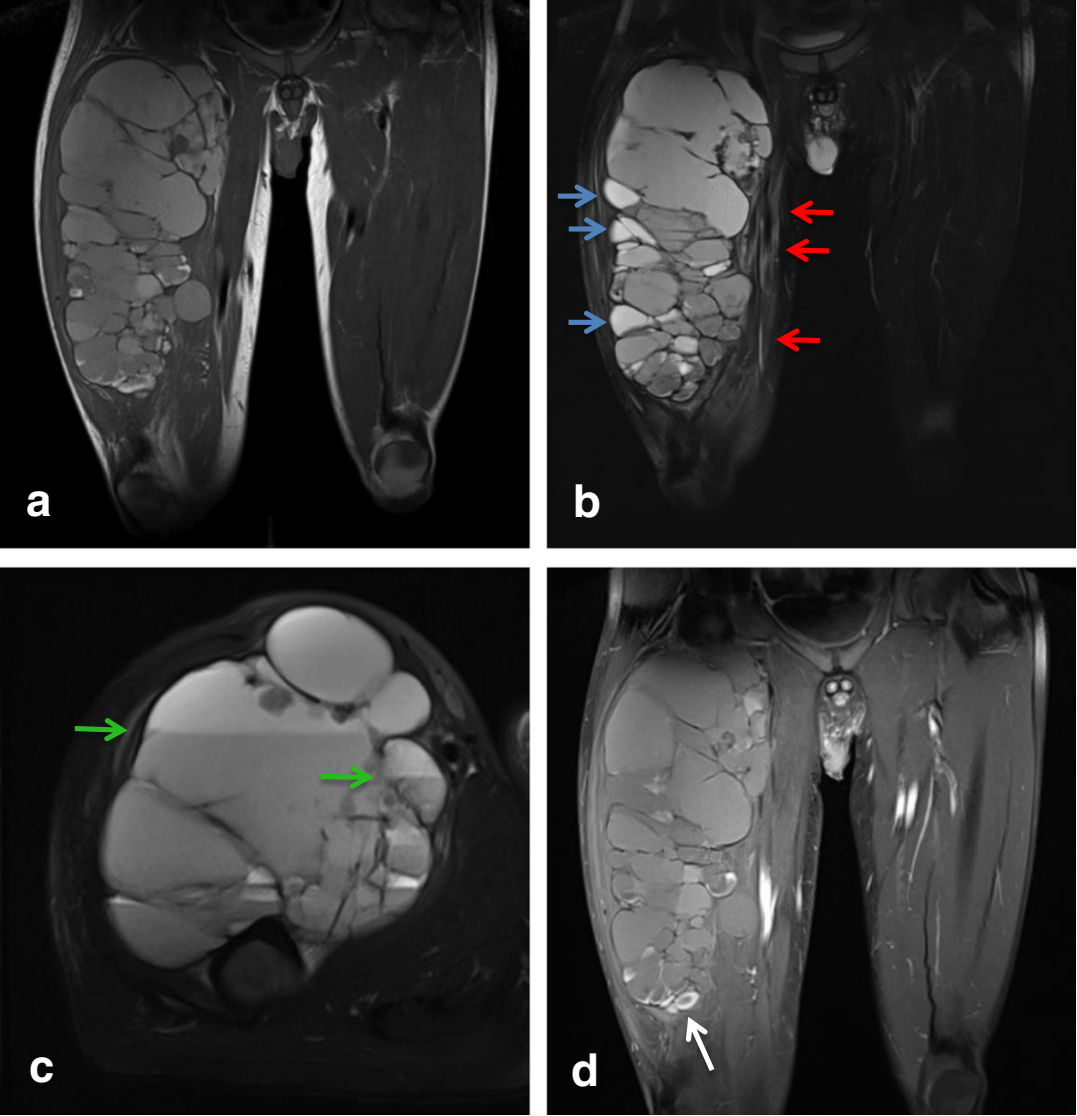
A 34-year-old man diagnosed with angiomatoid fibrous histiocytoma (case 6): (**a**) T1-weighted spin echo, (**b**, **c**) T2-weighted spin echo, and (**d**) contrast-enhanced images. A 163 × 130 × 303-mm painless mass is present in the vastus intermedius of the right thigh. The lesion is heterogeneously iso-hyperintense on T1 WI and presents with a multilocular area, cystic area (*blue arrows*), fluid–fluid level (green arrows), pseudocapsule, and peritumoral edema (*red arrows*) on T2 WI. A contrast-enhanced image shows partial gadolinium enhancement (*white arrows*)

Some groups have reported the MRI features of AFH, including cystic areas, pseudocapsules, peritumoral edema, enhancement, and fluid–fluid level [7–13]. On the basis of these findings, we evaluated the imaging characteristics of each case (Table 2, Figs. 3, 4 and 5). Almost all of the lesions were homogeneously or heterogeneously isointense to muscle on T1 WI and heterogeneously hyperintense on T2 WI. All cases, excluding case 7, presented variegated gadolinium-enhanced imaging. Additionally, pseudocapsule and multilocular findings were obtained in five cases and peritumoral edema was noted in six cases. We confirmed the cystic area and fluid–fluid level in two cases each. Based on MRI images, the initial diagnoses were hemangioma/arteriovenous malformation in two cases and hematoma, synovial sarcoma, and myxofibrosarcoma in one case each. No case was initially diagnosed as AFH.

### Histopathological diagnosis

No needle biopsy confirmed the diagnosis of AFH. Four of the seven cases were initially misdiagnosed as other soft tissue tumors (cases 2, 3, 5, and 6): these diagnoses included myxoid liposarcoma, Ewing’s sarcoma, myxofibrosarcoma, and synovial sarcoma. Case 4 was initially diagnosed as a spindle cell tumor. The diagnoses of these five cases (cases 2–6) were amended to AFH after pathological review or additional wide resection. Only two cases were correctly diagnosed as AFH by initial excisions (Table 3).

### Treatments and outcomes

The treatments and outcomes of AFH are presented in Table 3. Cases 2, 4, and 5 underwent wide excision in the first treatment. Case 1 was diagnosed as AFH after an excisional biopsy. Thereafter, we performed additional wide excision at our institution. Because cases 3 and 6 were diagnosed as Ewing’s sarcoma and synovial sarcoma, respectively, at their former institutions, these patients were administered chemotherapy (case 3, vincristine, doxorubicin, ifosfamide, and actinomycin-D; case 6, doxorubicin and ifosfamide) and wide excision. In case 7, only a marginal resection was performed. For five patients (cases 1, 2, 4, 5, and 7), no further local recurrence or distant metastasis was observed on the last day of follow-up. Case 3 had local recurrence and lymph node metastases at six months after the operation. Therefore, we performed wide re-excision and lymph node dissection. This patient has had no local recurrence or metastasis during the two years following the operation. The case 6 patient developed local recurrence twice and multiple lung metastases after wide resection. The patient was administered chemotherapy (gemcitabine and docetaxel); unfortunately, this patient died four years after initial treatment despite the induction of systemic chemotherapy.

## Discussion

Classified as an intermediate tumor using the WHO classification, AFH rarely metastasizes [2]. It generally follows an excellent clinical course overall. It frequently occurs in children and young adults in soft tissues, forming a well-circumscribed subcutaneous nodule on the extremities, head, neck, and trunk. No well-organized clinical reports exist for AFH because it is a very rare soft tissue tumor.

Although patients with AFH typically present with a subcutaneous soft tissue lump, Costa and Weiss et al. reported that 18% of tumors invaded deep structures such as skeletal muscle [3]. In our series, three cases (43%) were in deep lesions. According to previous reports, pain and tenderness were rarely encountered in AFH patients [1, 6, 14]. In our series, only one case (case 3) had a painful mass. In addition, AFH often occurs in children and young adults [1, 2]. However, three of seven patients examined in the present study were older than 30 years.

On MRI examination, AFH shows homogeneously hypointense lesions on the T1 WI and heterogeneously hyperintense lesions on the T2 WI [12]. The pattern of gadolinium enhancement is a variegated internal and nodular peripheral appearance. In addition, some groups reported that AFH shows cystic areas, pseudocapsules, hemosiderin, and fluid–fluid levels on MRI [7–13]. Martinez et al. reported the double rim sign and invasive pattern as novel MRI findings for AFH [15]. The double rim sign refers to the presence of a rim of high signal intensity (RHS) and an adjacent rim of low signal intensity (RLS), which can be observed on both T2-weighted and post-contrast images. The invasive pattern presents as irregular infiltrating peritumoral strings of high signal intensity on T2-weighted and post-contrast images. We believe that these signs were present in the pseudocapsule, peritumoral edema, and enhancement of our series. However, these two typical views were observed only in two cases (cases 3 and 5). Consequently, the MRI findings of AFH are nonspecific. Because of its radiological features and indolent clinical behavior [14], it is often mistaken for a benign condition such as a hematoma or hemangioma, potentially leading to inappropriate treatments. Indeed, in our series, three cases were misdiagnosed from initial MRI findings as a hematoma or hemangioma. Consequently, histopathological examination is extremely important to reach a definitive diagnosis.

The characteristic histological features of AFH have been well described [2]. These features include the following: (i) multinodular growth of myoid spindled or histiocytoid cells with a distinctive syncytial appearance, (ii) pseudoangiomatous spaces filled with blood and surrounded by tumor cells, (iii) a thick fibrous pseudocapsule with prominent hemosiderin deposition, and (iv) peritumoral lymphoplasmacytic cuffing with occasional germinal center formation. However, AFH can display a wide morphological spectrum. One or more of the above-described histological findings might be lacking. Nevertheless, multinodular growth of myoid spindle cells is consistent. Devoting attention to this pattern and cytology is crucial for accurate diagnosis. Unusual morphological features that have been reported in a small number of AFHs include clear cells, small cells with scanty cytoplasm resembling Ewing’s sarcoma, and rhabdomyoblast-like cells [2, 16, 17]. In addition, a myxoid variant has been established recently in which the tumors exhibit reticular growth in a prominent myxoid background. The diagnosis of myxoid AFH can be particularly challenging. Differential diagnosis includes other myxoid tumors such as low-grade fibromyxoid sarcoma, extraskeletal myxoid chondrosarcoma, and myxoid liposarcoma [18]. Although there are no entirely specific immunohistochemical markers for AFH, approximately half of the tumors express desmin. Epithelial membrane antigen expression is also characteristic of AFHs. In addition, AFHs can be uncommonly positive for other myoid markers such as smooth muscle actin, calponin, or, rarely, h-caldesmon, but skeletal muscle markers such as myogenin or MyoD1 are consistently negative [19]. In our series, aside from two cases (cases 1 and 7), none were diagnosed correctly as AFH from the initial biopsies. Cases 2 and 6 were misdiagnosed even after excision.

Recent reports have noted that molecular analyses are useful ancillary diagnostic techniques for AFH. These analyses include fluorescence in situ hybridization (FISH) to detect the rearrangement of EWSR1 or FUS and reverse transcription-polymerase chain reaction (RT-PCR) to elucidate EWSR1-CREB1, EWSR1-ATF1, or FUS-ATF1 fusion transcripts [16, 17, 20]. Tanas et al. reported that 76% of assessed AFH were shown by FISH to harbor EWSR1 rearrangement [20]. Thway et al. showed that both FISH and RT-PCR are equally reliable for facilitating an AFH diagnosis because one technique can identify the cases that the other method misses [21]. The detection of EWSR1 rearrangement by FISH was indeed helpful for reaching the correct diagnosis in our series, including five cases (cases 2–6) to which incorrect labeling was assigned initially. In addition, CREB1 gene rearrangement further supported the diagnoses in 2 cases (cases 3 and 6) that showed unexpected aggressive courses. We believe that molecular methods are sometimes necessary for the diagnosis of AFH.

A summary and comparison of the clinical outcomes of AFH cases described in earlier reports are presented in Table 4. Although some groups reported a favorable prognosis of AFH after treatment [3, 22, 23], Enzinger et al. reported that 63% and 21% of patients had local recurrence and metastasis, respectively, and that 12% of patients had died of the disease. In a study by Pettinato et al. [22], the prognoses were not good: recurrence occurred in 25% of cases, metastasis occurred in 5% of cases, and death occurred in 5% of cases. However, some reports published before the establishment of molecular analyses might have included malignant neoplasms other than AFH. In our series, five patients remained alive and well without recurrence or metastasis after resection. However, two patients (29%) had tumor recurrence and metastasis, one of whom died from disease progression. Notably, the diagnosis of AFH in both of these aggressive cases was confirmed by FISH on a molecular level for the presence of rearrangements of both EWSR1 and CREB1. Tamas et al. reported that the influence of the EWSR1-CREB1 fusion gene on prognosis was not clear [24]. In the AFH cases in which the EWSR1-CREB1 fusion gene was detected, only one recurrence case was reported [16]; there were no reported deaths. Case 6 may be the first fatal case in which EWSR1-CREB1 was detected. Therefore, the clinical outcome of our cases was unexpectedly worse than that of others described in the literature. Additionally, we administered systemic chemotherapy in case 6, but it was ineffective. Our results suggest that AFH patients may follow an unfortunate course that can rarely be predicted.

**Table 4:** Literature review

Author	No. of cases	Average age (yr range))	Recurrence cases (%)	Metastasis cases (%)	Death cases (%)	Follow-up (mo. (range))
Enzinger [1]	24	13 (5–25)	15 (63)	5 (21)	3 (12)	36 (12–240)
Costa and Weiss et al. [3]	108	17 (2–70)	11 (12)	4 (4)	1 (1)	63 (5–189)
Pettinato et al. [22]	20	13.4 (3–42)	5 (25)	1 (5)	1 (5)	NA
Fanburg et al. [19]	158	20 (2–71)	2 (2)	1 (1)	0	6 (12–276)
Hasegawa et al. [23]	4	22.7 (0.5–54)	1 (25)	0	0	11 (84–204)
Chen et al. [16]	8	48 (22–65)	1 (12.5)	0	0	21 (3–78)
Shi et al. [25]	21	26.9 (8–83)	2 (9.5)	0	0	48 (4–148)
Wilk et al. [26]	9	24 (3–67)	0	NA	NA	75 (30–132)
Our series	7	26.4 (8–50)	2 (29)	2 (29)	1 (14.3)	35.1 (5–61)

*NA* not applicable

Costa et al. reported that an irregular tumor border as well as a head and neck location were associated with local recurrence, and the depth of the tumor was correlated with subsequent local and distant metastasis [3]. Furthermore, they showed that the mitotic activity, extreme pleomorphism, inflammatory response, tumor size, patient age, and adjuvant therapy were not correlated with the clinical behavior. In other studies, the degree of mitotic activity and atypia showed no correlation with the risk of recurrence. There have been no reports showing that genetic factors or immunohistochemical profiles are related to clinical behavior. In our small number of cases, we detected no definite correlation between clinical characteristics and prognosis. Nevertheless, we did note several features that might be relevant in the two cases that showed an aggressive course (cases 3 and 6). Specifically, the primary tumor in case 3 was the only one in our series that presented as a painful mass, and it exhibited an unusual small round cell morphology with marked mitotic activity, although its recurrence showed the classic spindle cell histology of AFH. Case 6 manifested as a large mass in a deep location, and its recurrence demonstrated focally increased nuclear atypia and pleomorphism compared to the classic morphology of primary tumors. Interestingly, these pleomorphic tumor cells harbored an increased (up to 12 copies) number of rearranged EWSR1 genes in contrast to a signal copy in non-pleomorphic areas. We suspect that it may be worthwhile to investigate these features to see if they predict local recurrence and distant metastasis.

## Conclusions

Not only is AFH a rare soft tissue neoplasm, but its diagnosis is often difficult because of the lack of specific clinical symptoms and radiological and histopathological features. In our series, AFH occurred in older patients and was found in deep lesions. Although it appears that patients with AFH have excellent prognoses, the rates of local recurrence and metastasis might be higher than initially expected. Surgeons must therefore be aware of AFH and include it in clinical, radiological, and histological differential diagnoses.

## Abbreviations

AFH: Angiomatoid fibrous histiocytoma
FISH: Fluorescence in situ hybridization
MRI: Magnetic resonance imaging
RT-PCR: Reverse transcription-polymerase chain reaction
